# Liposomal Curcumin is Better than Curcumin to Alleviate Complications in Experimental Diabetic Mellitus

**DOI:** 10.3390/molecules24050846

**Published:** 2019-02-27

**Authors:** Adriana Elena Bulboacă, Alina S. Porfire, Lucia R. Tefas, Paul Mihai Boarescu, Sorana D. Bolboacă, Ioana C. Stănescu, Angelo Corneliu Bulboacă, Gabriela Dogaru

**Affiliations:** 1 Department of Pathophysiology, Iuliu Haţieganu University of Medicine and Pharmacy Cluj-Napoca, Victor Babeş Str. No. 4–6, 400012 Cluj-Napoca, Romania; adriana.bulboaca@umfcluj.ro; 2 Department of Pharmaceutical Technology and Biopharmaceutics, Iuliu-Haţieganu University of Medicine and Pharmacy Cluj-Napoca, Victor Babeş Str., No. 43, 400012 Cluj-Napoca, Romania; alinatuns@yahoo.com (A.S.P.); lucia.tefas@yahoo.com (L.R.T.); 3 Department of Medical Informatics and Biostatistics, Iuliu Haţieganu University of Medicine and Pharmacy Cluj-Napoca, Louis Pasteur Str., No. 6, 400349 Cluj-Napoca, Romania; 4 Department of Neurology and Pediatric Neurology, Iuliu Haţieganu University of Medicine and Pharmacy Cluj-Napoca, Victor Babeş Str., No. 43, 400012 Cluj-Napoca, Romania; ioanastane@yahoo.com (I.C.S.); angelo.bulboaca@yahoo.com (A.C.B.); 5 Department of Physical Medicine and Rehabilitation, Iuliu Haţieganu University of Medicine and Pharmacy Cluj-Napoca, Victor Babeş Str., No. 43, 400012 Cluj-Napoca, Romania; dogarugabrielaumf@gmail.com

**Keywords:** curcumin, oxidative stress, diabetes mellitus (DM), streptozotocin (STZ), nitric oxide (NOx), malondialdehyde (MDA), catalase, matrix metalloproteinases (MMP)

## Abstract

Curcumin (CC) is known to have anti-inflammatory and anti-oxidative properties and has already been tested for its efficiency in different diseases including diabetes mellitus (DM). New formulations and route administration were designed to obtain products with higher bioavailability. Our study aimed to test the effect of intraperitoneal (i.p.) administration of liposomal curcumin (lCC) as pre-treatment in streptozotocin(STZ)-induced DM in rats on oxidative stress, liver, and pancreatic functional parameters. Forty-two Wistar-Bratislava rats were randomly divided into six groups (seven animals/group): control (no diabetes), control-STZ (STZ-induced DM —60 mg/100g body weight a single dose intraperitoneal administration, and no CC pre-treatment), two groups with DM and CC pre-treatment (1mg/100g bw—STZ + CC1, 2 mg/100g bw—STZ + CC2), and two groups with DM and lCC pre-treatment (1 mg/100g bw—STZ + lCC1, 2 mg/100g bw—STZ + lCC1). Intraperitoneal administration of Curcumin in diabetic rats showed a significant reduction of nitric oxide, malondialdehyde, total oxidative stress, and catalase for both evaluated formulations (CC and lCC) compared to control group (*p* < 0.005), with higher efficacy of lCC formulation compared to CC solution (*p* < 0.002, excepting catalase for STZ + CC2vs. STZ + lCC1when *p* = 0.0845). The CC and lCC showed hepatoprotective and hypoglycemic effects, a decrease in oxidative stress and improvement in anti-oxidative capacity status against STZ-induced DM in rats (*p* < 0.002). The lCC also proved better efficacy on MMP-2, and -9 plasma levels as compared to CC (*p* < 0.003, excepting STZ + CC2 vs. STZ + lCC1 comparison with *p* = 0.0553). The lCC demonstrated significantly better efficacy as compared to curcumin solution on all serum levels of the investigated markers, sustaining its possible use as adjuvant therapy in DM.

## 1. Introduction

Diabetes mellitus (DM) is defined by hyperglycemia resulting from defects in insulin secretion, insulin action, or both, classified as type 1 diabetes (type 1 DM), type 2 diabetes mellitus (type 2 DM), other specific types of diabetes mellitus, and gestational diabetes [1]. In type 1 DM an autoimmune mechanism, which disrupts insulin production by beta cells is observed, while the type 2 DM occurs due to insulin resistance coupled with insufficient production of insulin [1]. Animal models for testing various new therapies frequently use experimental type 1 diabetes and range from models with spontaneously developing autoimmune diabetes to chemical ablation of the pancreatic beta cells [2]. Chemical induction of DM by streptozotocin (STZ) administration is one of the most frequently used animal models for experimental type 1 diabetes mellitus. Streptozotocin [2-deoxy-2-(3-(methyl-3-nitrosoureido)-d-glucopyranose] is synthesized by *Streptomyces achromogenes* [3]. After i.p. or i.v. administration, STZ, as a pancreatic β-cell-specific cytotoxin, enters pancreatic β cells via the Glut-2 transporter inducing DNA alkylation and fragmentation [4]. Subsequently, the fragmented DNA activates reparative enzymes that deplete the cells in ATP [5,6]. As a result of ATP depletion, dephosphorylation provides more substrates molecules for increasing oxidative stress [4]. During this process, the presence of the *N*-methyl-*N*-nitrosourea side chain can increase the nitro-oxidative stress due to releasing of nitric oxide (NO) [7].

Consequently to the oxidative stress induced by STZ administration, an inflammatory reaction follows, and the first cells type that contributes to cellular response and infiltrates the islet cells are the macrophages [8]. The production of cytokine is related to the development of diabetes [8]. Due to its cytotoxic proprieties for beta islet cells, STZ can induce DM without involving the autoimmune mechanism [9]. The cytotoxicity of STZ is presumed to be mediated by reactive oxygen species (ROS), reactive nitric oxide species, and induction of inflammatory responses [7]. Furthermore, hyperglycemia leads to more elevated ROS, which has an essential effect on some metabolic pathways and promotes diabetic vascular disease [10].

Along with oxidative stress and inflammation, matrix metalloproteinases (MMP) can play an essential role in DM pathogenesis and its vascular complications [11]. MMPs, a family of zinc-dependent endopeptidases, degrade extracellular matrix (ECM) and contribute to extracellular matrix (ECM) remodeling [12]. MMPs are inhibited by a family of proteins called tissue inhibitors of MMPs (TIMPs) [12]. Their role in ECM degradation and remodeling was previously studied for cardiac muscle damage due to myocardial infarction [13]. It has been hypothesized that MMP-2, a member of the gelatinase family of proteases, which also plays a vital role in myocardial ischemia-reperfusion injury [14], may also have biological functions such as proteolysis of cytoskeletal proteins, increasing oxidative stress. MMP inhibition can lead to attenuation of tissue damage [15]. Elevated MMP-2 had been reported to be implicated in the development of experimental diabetes mellitus in rats, and an MMP inhibitor, PD166793, reduces blood glucose [16]. In vitro studies showed that MMP-2 expression and activity is increased in rat pancreatic beta cell line INS-1 treated with advanced glycation end-products (AGE), and is accompanied by increased reactive oxygen species levels [17]. Moreover, treatment with *N*-acetylcysteine (NAC), which reduced oxidative stress, inhibited MMP-2 expression and activity and partially reversed cell apoptosis induced by AGE [17]. Type IV collagenases, MMP-2 (72kDa), and gelatinase-B, MMP-9 (92kDa) are most likely to contribute to the microvascular complications of diabetes mellitus, especially in diabetic retinopathy due to capillary cell apoptosis mechanism as previously reported on animal models [18,19].

Therefore, antioxidants therapies could be important for beta cells morphological and functional preservation, reducing free radicals synthesis from injured β cells. As a new therapeutic option, various antioxidants therapies are considered a valuable alternative for β cell protection. Vitamins (A, E or C vitamins), medicinal plants extracts (various flavonoids), enzymatic cofactors (folic acid, vitamins B1, B2, B6, B12), and antioxidant minerals (copper, zinc, selenium, and manganese) were studied [20]. Therapies targeting cytokines [21], oxidative stress molecules (serum malondialdehyde, glutathione peroxidase, and superoxide dismutase) [22], and NO [23] tend to display efficiency, indicating their beneficial role in β cell preservation.

Curcumin (derived from turmeric plant) is a natural antioxidant and anti-inflammatory agent that previously demonstrated its ability to reduce the effect of STZ on oxidative stress, in experimental diabetes in rats, when given by oral routes [24,25]. Curcumin pre-treatment can also mediate functional regulation of adrenergic receptors and induce the modulation of key cell signaling molecules that result in improving insulin gene expression, and insulin secretion [26]. The difficulties of clinical translation of experimental studies result from its limited oral bioavailability [27]. Plasma concentration of curcumin in oral administration and its tissues level were found to be low, as long as poor absorption, rapid metabolism, and rapid systemic elimination [28,29]. Several studies reported that the oral bioavailability of curcumin could be improved by the use of adjuvant piperine which can interfere with glucuronidation [30], curcumin nanoparticles [31,32], and structural analogs of curcumin [33]. Ganugula et al. reported that nano-curcumin (particle size of 300 nm, p.o. administration) could prevent STZ-induced inflammation and apoptosis in pancreatic islets cells, reflected in the reduction of glucose level, pro-inflammatory cytokines, and oxidative stress [34]. Activation of MMPs is an early event in oxidative stress damage of different tissues such as brain [35], heart [36] or lungs [37]. Therefore, their plasma concentration could be a good and a rapid indicator for β cell destruction due to STZ-induced diabetes mellitus in an experimental model.

Our study is aimed to compare the effect of curcumin by intraperitoneal route administration as a pre-treatment as two forms, namely curcumin solution and liposomal curcumin, in experimental diabetes induced by STZ in rats. We compared the difference between curcumin solution and liposomal curcumin administration, regarding the influence on the liver and pancreatic functional parameters (serum transaminases, glycemia, MMP-2, and MMP-9), and oxidative stress/antioxidant status parameters in plasma.

## 2. Results

The inducement of diabetes was successfully achieved, and all rats were included in the final analysis. The oxidative stress parameters were significantly (Mann-Whitney test *p* < 0.002) increased (Table 1) after diabetes was induced, as well as glycemia, hepatic enzymes, and matrix metalloproteinases (MMP-2 and MMP-9). On the other hand, the inducement of diabetes mellitus led to a significant decrease in antioxidant capacity quantified by TAC and catalase (Table 1; Mann-Whitney test *p* < 0.002).

**Figure 1:**
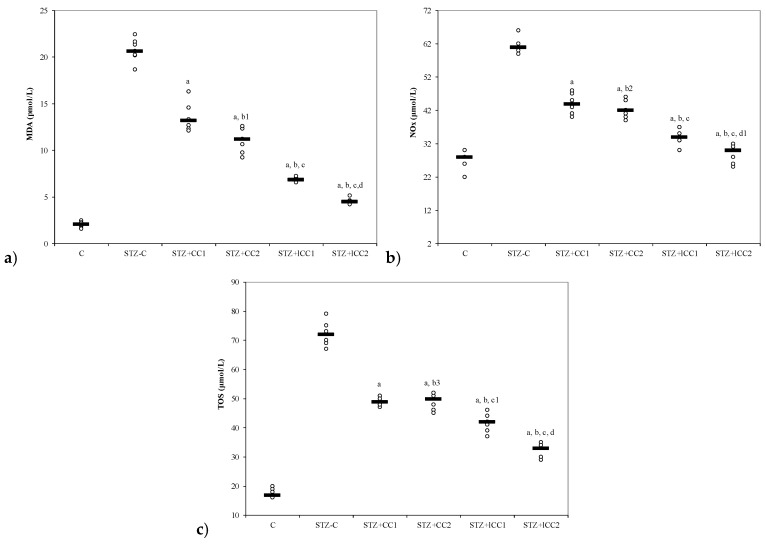
Variability of oxidative stress intensity by groups: (**a**) MDA (malondialdehyde) and (**b**) NOx (nitric oxide). C = control; STZ-C = streptozotocin control; STZ + CC1 = STZ and 1 mg/100 g bw CC as pre-treatment; STZ + CC2 = STZ and 2 mg/100 g bw CC as pre-treatment; STZ + lCC1 = STZ and pre-treatment with 1 mg/100 g bw lCC; STZ + lCC2 = STZ and pre-treatment with 2 mg/100 g bw lCC. ^a^ *p*-values < 0.002 as compared to STZ-C group; ^b^ *p*-values < 0.002 as compared to STZ + CC1 group excepting ^b1^ 0.0073, ^b2^ 0.2502, and ^b3^ 0.6547; ^c^ *p*-values< 0.002 as compared to STZ + CC2 group excepting ^c1^ 0.0040; ^d^ *p*-values < 0.002 as compared to STZ + lCC1 group excepting ^d1^ 0.0181. (**c**) TOS (total oxidative status).

**Figure 2:**
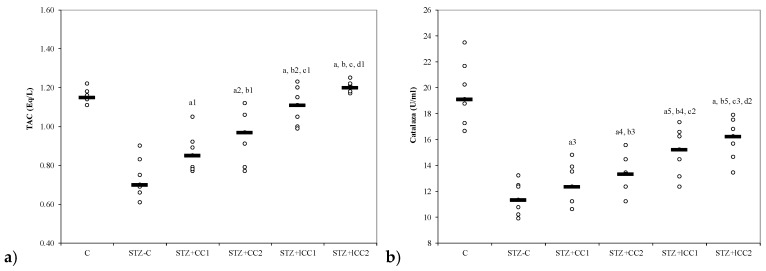
Variability of antioxidant capacity of plasma by groups: (**a**) total antioxidant capacity (TAC) and (**b**) catalase. C = control; STZ-C = streptozotocin control; STZ + CC1 = STZ and 1 mg/100 g bw CC as pre-treatment; STZ + CC2 = STZ and 2 mg/100 g bw CC as pre-treatment; STZ + lCC1 = STZ and pre-treatment with 1 mg/100 g bw lCC; STZ + lCC2 = STZ and pre-treatment with 2 mg/100 g bw lCC. ^a^ *p*-values < 0.002 as compared to STZ-C group and ^a1^ 0.0350, ^a2^ 0.0088, ^a3^ 0.2502, ^a4^ 0.0409, and ^a5^ 0.0060, respectively; ^b^ *p*-values < 0.002 as compared to STZ + CC1 group excepting ^b1^ 0.2248, ^b2^ 0.0049, ^b3^ 0.4433, ^b4^ 0.0253, and ^b5^ 0.0088; ^c^ *p*-values < 0.002 as compared to STZ + CC2 group excepting ^c1^ 0.0253, ^c2^ 0.0845, and ^c3^ 0.0060; ^d^ *p*-values as compared to STZ + lCC1 group: ^d1^ 0.0476, ^d2^ 0.2502.

**Figure 3:**
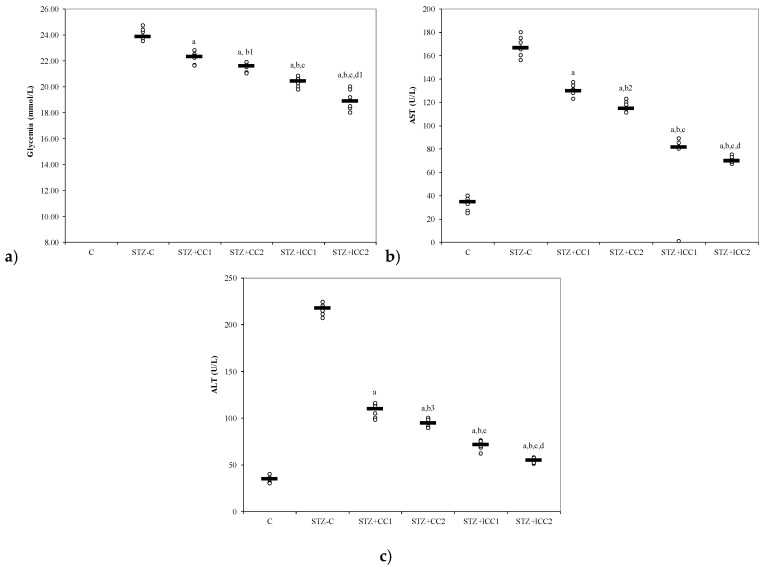
Variability of (**a**) glycemia, (**b**) AST (aspartate aminotransferase) and (**c**) alanine aminotransferase (ALT) by groups. C = control; STZ-C = streptozotocin control; STZ + CC1 = STZ and 1 mg/100 g bw CC as pre-treatment; STZ + CC2 = STZ and 2 mg/100 g bw CC as pre-treatment; STZ + lCC1 = STZ and pre-treatment with 1 mg/100 g bw lCC; STZ + nCC2 = STZ and pre-treatment with 2 mg/100 g bw lCC. ^a^ *p*-values < 0.002 as compared to STZ-C group; ^b^ *p*-values < 0.002 as compared to STZ + CC1 group excepting ^b1^ 0.0152, ^b2^ 0.0027, and ^b3^ 0.0060; ^c^ *p*-values < 0.002 as compared to STZ + CC2 group excepting ^c1^ 0.0040; ^d^ *p*-values < 0.002 as compared to STZ + lCC1 group excepting ^d1^ 0.0040.

**Figure 4:**
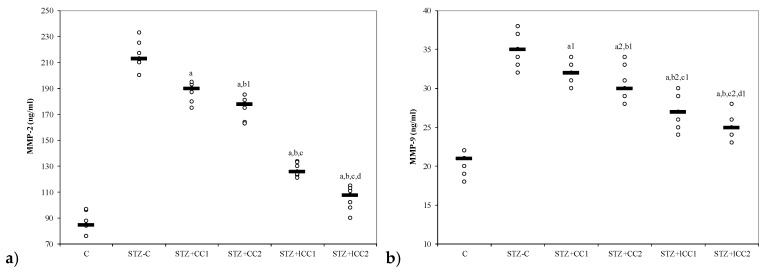
Variability of matrix metalloproteinases (MMP) by groups: (**a**) MMP-2, (**b**) MMP-C = control; STZ-C = streptozotocin control; STZ + CC1 = STZ and 1 mg/100 g bw CC as pre-treatment; STZ + CC2 = STZ and 2 mg/100 g bw CC as pre-treatment; STZ + lCC1 = STZ and pre-treatment with 1 mg/100 g bw lCC; STZ + lCC2 = STZ and pre-treatment with 2 mg/100 g bw lCC. ^a^ *p*-values < 0.002 as compared to STZ-C group excepting ^a1^ 0.0181 and ^a2^ 0.0088; ^b^ *p*-values as compared to STZ + CC1 group of 0.0017 excepting ^b1^ 0.1797 and^b2^ 0.0027; ^c^ *p*-values as compared to STZ + CC2 group < 0.002 excepting ^c1^ 0.0553 and ^c2^ 0.0027; ^d^ *p*-value as compared to STZ + lCC1 group of 0.0017 excepting ^d1^ 0.0845.

The oxidative stress was significantly reduced by curcumin pre-treatment in both formulations (CC and lCC), compared with STZ-control group (STZ-C) (*p*-values < 0.0002), with better results for lCC as compared to CC, for both doses (*p*-values <0.005) (Figure 1, Figure 2, Figure 3 and Figure 4). The antioxidant capacity evaluated by TAC was proved not to be significantly different as compared to the controls at α = 0.005 for both doses of liposomal curcumin pre-treatment (STZ + lCC1: *p* = 0.3067 for TAC; STZ + lCC2: *p* = 0.0253). The values of catalase were significantly different for lCC pre-treatment groups as compared to control group (*p* = 0.004 for the lCC1 group and 0.0127 for the lCC2 group). The values of MMP-2 and MMP-9 closest to the control group were obtained in the group with the highest dose of lCC (Table 1, STZ + lCC2: *p* = 0.0040 for MMP-2 and *p* < 0.002 for MMP-9).

The Kruskal-Wallis ANOVA test identified significant differences between the groups with diabetes and different pre-treatments for all evaluated parameters (*p*-values < 0.0001). The post-hoc analysis identified significant differences in most of the cases with better protection as the curcumin dose increases (for both forms of curcumin) and significantly higher protection when liposomal curcumin was used (Figure 1, Figure 2, Figure 3 and Figure 4).

## 3. Discussion

Liposomal curcumin administration proved to be more effective compared to curcumin solution, with significant improvement of functional liver and pancreatic markers, as well as oxidative/antioxidative parameters, in STZ-induced diabetes mellitus in rats.

From our knowledge, the effects of intraperitoneal administration of liposomal curcumin in diabetes induced by streptozotocin in rats have not been studied before. In our study, β cell toxicity and liver cell damage induced by oxidative stress, developed after STZ administration, were recognized by the alterations of the functional liver and pancreatic markers, and oxidative/antioxidative stress parameters (Table 1, Figure 1, Figure 2, Figure 3 and Figure 4). Biochemical alterations included the increase of glycemia, and an increase in liver enzymes levels AST and ALT (Table 1, Figure 3) as a marker for pancreatic metabolic function and liver damage, respectively. The comparison of groups with curcumin pre-treatment showed significant differences in regards to glycemia and hepatic enzyme activities with better improvements after lCC administration (Figure 3). The curcumin effect as a pre-treatment, in our experimental groups, is based on the potential ability of curcumin to improve glycemia and transaminases, previously demonstrated for oral curcumin administration [24]. Curcumin had also an effect on oxidative stress parameters, including the increase of NO, MDA, and TOS levels and on antioxidant status modified by the increase of catalase activities, and TAC levels (Figure 1 and Figure 2). Excessive production of reactive oxygen species (ROS) and/or their inadequate neutralization by antioxidants, leads to damage in the cell membrane and the vessel wall, contributing to the initiation and progressing of diabetic complications [38].

On the other hand, hyperglycemia itself, produced by STZ in our study, can contribute to an increase in oxidative stress [39]. The overproduction of the nitric oxide (NO), by activated immunocompetent cells, is supposed to be one of the possible pathophysiological mechanisms of β cell damage induced by streptozotocin [40]. These results emphasize that curcumin may modulate the molecular pathways involved in the inflammation and the immune response, being a valuable nutritional compound for supplement therapies in various diseases [41]. 

### 3.1. Beta Cells and Hepatic Cells Protective Effect of Liposomal Curcumin

Despite its high metabolic activity, pancreatic tissue possesses a low anti-oxidative capacity compared to other tissues, being more vulnerable to oxidative stress that can lead to pancreatic beta cell death [42]. The protective effect of curcumin on pancreatic metabolic function is revealed by decreasing of plasma glucose after curcumin administration in all the treatment groups compared to the control group with a more significant effect of liposomal curcumin (Figure 3). β Cell protection by oral curcumin administration as a pre-treatment in experimental DM-induced by STZ was previously reported [24,34,43]. Both forms (solution and liposomal curcumin) were able to decrease plasma glucose concentration with better results for liposomal curcumin formula (Figure 3) which also reported by previous studies [34]. Intraperitoneal administration of multiple doses of curcumin solution did not cause any apparent toxicity in diabetic mice, delayed the disease onset and improved the glucose tolerance test used as an indicator for beta cells function [44]. Therefore, the effect of intraperitoneal administration of a single dose of liposomal curcumin can achieve the target for prevention or management of diabetes by the insulin-secreting beta cells function preservation.

In physiological conditions, the pro-oxidants like reactive nitrogen species and reactive oxygen species produced by the liver in aerobic metabolism can be neutralized by antioxidant systems [45]. Previous studies related to liver damage induced by toxic mechanisms (carbon tetrachloride [46,47] and ethyl alcohol-induced liver cells injury [48,49]) showed the importance of oxidative stress in liver damage. The oxidative stress is considered a molecular defense mechanism against the rapidly occurring hepatic cells damage and is related to activation of specific stress responses genes and activation of different members of heat shock proteins family (hsp70, hsc73, grp78) [50]. Furthermore, the protective effect of curcumin on liver injury produced by endotoxemic shock, in a murine model, was previously demonstrated by Yun et al. [51]. In hepatic injury, enhancement of oxidative stress is also leading to an imbalance between oxidants and antioxidants, accompanied by hepatocytolysis and increasing of AST and ALT [52]. ALT is an important biomarker for hepatocellular injury [53]. Elevated levels of ALT due to liver damage, increased oxidative stress and associated inflammation were previously reported in experimental diabetes mellitus induced by STZ [54]. Our parameters, after STZ administration, show the same results (increasing the ALT and AST level in the group with STZ administration compared to the control group, Table 1, Figure 3). Curcumin administration in all groups significantly reduced the level of both transaminases, with better results in liposomal CC groups (Table 1, Figure 4). Antioxidant and hepatoprotective proprieties of curcumin administered by oral route had already been shown in other studies [24,55,56]. It was reported to decrease the production of cytokines, including TNF-α and TNF-β, by inhibiting NF-κB, and thus is likely to possess a protective effect on the liver by anti-inflammatory proprieties [57]. Curcumin can protect pancreatic β cells from cytokine-induced cell death by scavenging ROS and normalizing cytokine-induced NF-kappaB translocation (in vitro study) [58]. Curcumin can also improve liver histopathology in an early stage of ethanol-induced liver injury by reduction of oxidative stress (decreasing hepatic MDA and hepatocyte apoptosis), by inhibition of NF-*κ*B activation [59]. In hepatic injury, curcumin can act on different molecules implicated in hepatocytolysis such as matrix metalloproteinases (MMPs) [60], apoptotic pathway [61], and inflammatory cytokines [62,63]. A previous study has shown that pro-inflammatory cytokine concentrations in the serum and pancreas were raised in STZ-treated animals, but not in animals pre-treated with curcumin before STZ [58].

### 3.2. Effects of Liposomal Curcumin on Oxidative Stress Parameters

Our study’s results show that NO, MDA and TOs were significantly improved in groups with curcumin pre-treatment, compared to the control group (Table 1, Figure 1). The beneficial effects of the curcumin on oxidative stress parameters associated with liver damage were also described for non-alcoholic steatohepatitis, alcoholic liver disease, or other liver disorders related to various etiological factors [64,65]. Nitric oxide, a physiological vasodilator, as well as a contributor to oxidative stress, can regulate leukocyte recruitment [66]. NO produced by endothelial cells (eNO) prevents leukocyte rolling and adhesion to postcapillary venules, being a significant portion of endogenous NO production as well as much of the circulating levels of nitrite (NO_2_^−^) in the body [67]. Thus, physiological concentrations of NO inhibits inflammation, while its high levels, as seen in inflammatory conditions, produce harmful actions [68]. NO overproduction in diabetes mellitus may be converted to peroxynitrite radical acting as a pro-inflammatory molecule [69]. Our study group (group 2) demonstrated a significantly increased NO production after i.p. STZ administration, compared to the control group (group 1, Table 1). Previous studies demonstrated that diabetes mellitus induced by STZ in rats it is associated with increased NO production due to increased nitroxidative stress [70]. Different isoforms of NO synthase contribute to NO production, and in experimental diabetes induced by STZ, NO is increased due to increased mitochondrial synthesis [71]. The maximum induction in the level of mitochondrial NO synthesis was observed in pancreatic tissue, followed by the liver, kidney, and brain tissues [71]. Simultaneously, there is a vicious cycle because the excess NO induces oxidative stress in mitochondria, characterized by an increase in the level of protein carbonyls and a decrease in glutathione (GSH) molecules [72].

Consequently, the formation of reactive nitrogen species (RNS), such as peroxynitrite, can lead to the irreversible modification of cell’s proteins [73]. There are previous results that showed a decrease in NO production by reducing iNOS activity, after oral administration of curcumin, in experimental diabetes induced by STZ [74]. Intraperitoneal administration of Ccurcumin can also prove to ameliorate the increased NO synthesis in experimental diabetes in mice [44]. Our study proved that liposomal curcumin was able to reduce the plasma level of NO for both curcumin concentrations (Table 1, Figure 1).

The first stage of cellular damage mediated by ROS is peroxidation of the cell membrane components, especially lipids (lipid peroxidation) [75]. The MDA, a product of lipid peroxidation, is widely used as a biomarker for ROS-dependent tissues damage, resulting from enhancing of oxidative stress due to STZ administration [24,76]. Moreover, the reaction between MDA and cell membrane proteins changes its antigenic proprieties [77] and produces dysfunction of membrane activity with consequences on its integrity [78]. The increase of MDA in STZ induced diabetes mellitus in rats has been previously demonstrated [79,80]. Feeding curcumin could decrease MDA significantly as previously demonstrated [81,82]. Our results demonstrated that intraperitoneal administration of liposomal curcumin (both concentrations) had better results compared with curcumin (Table 1) regarding the MDA reduction, proving its better effect.

### 3.3. Effects of Liposomal Curcumin on Antioxidative Status Parameters

Catalase levels, a parameter showing plasma oxidative status was significantly improved in the groups with curcumin administration as a pre-treatment in DM (both formulas—solution and liposomal curcumin) induced by STZ (Table 1, Figure 2). Antioxidant enzymes such as catalase exist in all oxygen-metabolizing cells, to prevent cell damage caused by oxidative stress molecules and to provide a repair mechanism for oxidized components [39]. Hydrogen peroxide (H_2_O_2_) levels increased in oxidative stress are neutralized by reduction to H_2_O by catalase [83]. Previous results have demonstrated that the activity of the antioxidant enzymes SOD, catalase and GSH-Px can prevent oxidative stress [84]. Gerber and Rutter have shown that pancreatic β cells are less damaged by oxidative stress, under diabetic conditions, by increasing catalase activity [42]. The decrease of catalase levels has been previously reported in experimental diabetes induced by STZ in rats [85]. Our results showed a significant decrease of catalase after i.p. curcumin administration, with better results for liposomal curcumin formulations (Table 1, Figure 2). Xie et al. [86] and Assis et al. [87] reported similar results, i.e., a reduction of activity of antioxidant systems (glutathione peroxidase and catalase), by oral administration of curcumin in STZ-induced diabetic rats. Orally administered curcumin in diabetes induced by STZ in rats has been shown to improve the anti-oxidative status by enhancing the levels of superoxide dismutase, catalase, and glutathione peroxidase [30]. The co-administration of curcumin with a bioenhancer (piperine), to increase curcumin bioavailability, did not make curcumin any more efficient in regards to its antidiabetic and antioxidant effects [30]. Adding quercetin to orally administered curcumin can have a better effect on improving the glycemia and lipid profile in STZ-induced diabetes in rats [88]. TAC has shown no significant differences between study groups (with curcumin pre-treatment, Table 1, Figure 2), probably due to a different anti-oxidant system, as components of anti-oxidative status, that remained unchanged after these doses of i.p. curcumin administration.

### 3.4. Effect of Liposomal Curcumin on Plasma Matrix Metalloproteinases

Table 1 and Figure 4 show a significant decrease in plasma MMP-2 and MMP-9 in groups with curcumin administration compared to the control group. MMPs expression in various pathologies is strongly correlated with cell apoptosis [89]. Pancreatic β cell apoptosis is present in both type 1 and 2 DM [90]. Diabetes mellitus is believed to stimulate the production of several MMPs which contribute to macrovascular and microvascular complications such as coronary artery disease, peripheral arterial disease, stroke, nephropathy, neuropathy, and retinopathy [91]. Experimental DM in rodents is associated with β cell apoptosis that leads to hyperglycemia, the macrophage proinflammatory cytokine (IL-1β in combination with IFN-γ and TNF-α), playing an essential role in β cell dysfunction and death [92]. Hyperglycemia, directly or indirectly (e.g., via oxidative stress or advanced glycation products) increases MMP expression and activity [91]. There is also evidence that both type 1 and type 2 DM are associated with systemic inflammation, and activated leukocytes can release substances that mediate endothelial damage and vascular destabilization (e.g., matrix metalloproteinases) [93,94,95]. Singh et al. showed, in a study performed on pancreatic cancer patients, that, there was a significant correlation between blood and tissue expression of MMP-2 protein meaning that assessment plasma concentration of MMP (which is easy to transfer in clinical situations) can reflect their tissues level [96]. It is possible that increased oxidative stress contributes to increased expression of MMP-2 in pancreatic cells and consequently to apoptosis in pancreatic tissue. Increase in plasma levels of MMP-2 and MMP-9 were also demonstrated by clinical studies in patients with type 2 DM, and those increases were before the onset of DM complications (microangiopathy or macroangiopathy) [97,98].

Regarding one of the macrovascular complication of DM (atherosclerosis) and its contribution to hypertension pathogenesis, De Rosa et al. demonstrated that similar increased concentrations of MMP-2 and MMP-9 in pre-hypertensive and hypertensive patients with type 2 DM may indicate early changes in vascular extracellular matrix turnover which, over the time, leads to the increase in arterial stiffness [97]. Ebihara et al. also demonstrated that increased MMP-9 in patients without albuminuria, compared with those with albuminuria and diabetic nephropathy, as a microvascular complication, were similar [98]. Diabetic retinopathy, a microvascular complication of DM, is also reported to be associated with an increased level of MMP-2 and -9 in aqueous humor, which was correlated with protein concentration, being essential for retinal neovascularisation assessment [99]. In our experiments, curcumin treatment by i.p. administration reduced the intensity of oxidative stress, associated with STZ-induced DM in rats, and also the level of MMP-2, being in this way a valuable adjuvant treatment for reducing the vascular complication of DM. Early onset of this therapy, when the vascular remodeling process is still reversible, could be an essential option for DM vascular complications prophylaxis.

### 3.5. Study Limitations

The first limitation of our study is related to the experimental design applied. Only the glycemic levels, general measures of metabolic health, was used to evaluate the pancreatic function. The evaluation of endogenous insulin and/or glucose sensitivity is needed for a more reliable evaluation of the pancreatic damage. The evaluation of hepatic function by serum transaminase levels is the second limitation of our study. The protective effect of liposomal curcumin concerning liver damage need a more rigorous histopathological analysis to identify and quantify the apoptotic cells or to detect excessive deoxyribonucleic acid breakage in individual cells to sustain its efficacy as compared to the curcumin solution. The third limitation is related to the serum evaluation of MMP-2 and MMP-The levels of MMP-2 and MMP-9 in the pancreatic and/or liver tissues would be very informative and closely reflect the efficacy of liposomal curcumin as compared to curcumin solution.

## 4. Materials and Methods

Wistar-Bratislava albino male rats (from the animal Department of the Faculty of Medicine and Pharmacy Cluj-Napoca, Cluj-Napoca, Romania, weighing 200–250 g) were used for this experimental study. The animals were kept in polypropylene cages at constant temperature (24 ± 2 °C), 60 ± 5% humidity, and light-dark regime. The animals received unrestricted access to food (standard pellets from Cantacuzino Institute, Bucharest, Romania) and water. All the procedures made in this experimental study were approved by the Ethics Committee of Iuliu-Hațieganu University of Medicine and Pharmacy Cluj-Napoca (protocol approval no. 374/16.10.2018) and were in accord with the rules of European Convention for the Protection of Vertebrate Animals used for Experimental and other Scientific Purposes. Every effort was made to reduce animal suffering and reduce the number of animals used.

### 4.1. Experimental Design

Six groups with seven animals/group were formed by random allocation of rats in the groups. The interventions applied to each group are presented in Table 2.

Curcumin i.p. administration was 30 min before STZ administration, according to Porfire et al. [101]. After the STZ was dissolved in a freshly prepared 0.01 M citrate buffer (pH = 4.5) solution, a single dose of 60 mg/kg was administered by i.p. route. After 72 h, fasting plasma glucose was checked, and the animals with plasma glucose values of more than 200 mg/dL were considered diabetic and were included in the study [34].

Curcumin (CC), cholesterol and streptozotocin were purchased from Sigma-Aldrich Co (St Louis, MO, USA). 1,2-Dipalmitoyl-*sn*-glycero-3-phosphocholine (DPPC, ≥99% [TP-PC]) and *N*-(carbonylmethoxypolyethylenglycol-2000)-1,2-distearoyl-sn-glycero-3phosphoethanolamine (PEG-2000-DSPE, ≥98% [HPLC]) sodium salt were purchased from Lipoid GmbH (Ludwigshafen, Germany). Metalloproteinases assessment was made with a kit purchased from R&D Systems Quantikine (McKinley Place NE, MN, USA). All other chemicals were of analytical grade.

Liposomal-curcumin was encapsulated in long-circulating liposomes (LCL) at a concentration of 4.7 mg/mL, using the film hydration method with a lipid molar ratio 9.5:0.5:1 (DPPC:PEG-2000-DSPE:CHO) as previously described [102,103]. The proposed formulation had appropriate quality attributes for intravenous administration, such as monodisperse size around 140 nm and zeta potential about −50 mV. A Curcumin solution of the same concentration was prepared by dissolution in 96% (*v*/*v*) ethanol and further dilution with saline in order to observe if liposomal encapsulated curcumin had an increased therapeutic effect.

### 4.2. Glycemia, Hepatic Enzyme Activities, Oxidative Stress Parameters, and Metalloproteinases Measurements

In order to measure the pancreatic metabolic function, serum was separated and analyzed for carbohydrate metabolic changes by assessment of glucose (glycemia) after 12 h of fasting. Glycemia was used as a serum marker for pancreatic metabolic function regarding the changes in carbohydrate metabolism due to experimental diabetes mellitus. For hepatic cells destruction assessment, serum levels of aspartate aminotransferase (AST) and alanine aminotransferase (ALT) were measured by an automated technique (Vita Lab Flexor E, Spankeren, The Netherlands).

The oxidative stress parameters were assessed from blood samples collected from the retro-orbital plexus of each rat, two days after the single dose of STZ was administrated [22], under ketamine anesthesia (5 mg/kg body weight, i.p route) [104]. The animals were euthanized by cervical dislocation after the collection of the blood samples. Spectroscopic measurements assessed the oxidative stress parameters. A Jasco V-350 UV-VIS spectrophotometer (Jasco International Co, Ltd., Tokyo, Japan) was used for all measurements. Two groups of parameters were measured: a) parameters for oxidative stress intensity: total oxidative status (TOS), malondialdehyde (MDA) [105], and indirect assessment of nitric oxide synthesis (NOx) [106] and b) parameters for antioxidant capacity of plasma: total anti-oxidative capacity (TAC) [106], and catlase [107]. Metalloproteinases assessment was made with a Stat Fax 303 ELISA reader (Quantikine, McKinley Place NE, MN, USA), using a rat ELISA kit (Boster Biological technology, Pleasanton, CA, USA).

### 4.3. Analysis of Data

Statistica program (v. 8, Stat Soft. Inc., Tusla, OK, USA) was used to analyze the data. The Kruskal-Wallis ANOVA, adjusted at a significant level by the number of investigated groups (five groups, 0.005), was used to test the differences in the investigated markers. The Mann-Whitney test was used in post-hoc analysis when significant differences were identified by Kruskal-Wallis ANOVA test. The differences in the values of the markers by groups were plotted as individual values (circles) and the median (the line) as recommended by Weissgerber et al. [108].

## 5. Conclusions

The beneficial effects of the liposomal curcumin on oxidative/anti-oxidant stress parameters pancreatic metabolic function and liver function markers proved to be higher as compared to the curcumin solution. The liposomal curcumin administration has shown to improve the level of all investigated biochemical markers being closest to the values of the control group (no diabetes mellitus). Furthermore, significantly better results are obtained on all investigated parameters by liposomal curcumin pre-treatment as compared to curcumin solution, regardless of the dose used.

Decreasing the MMP-2 and MMP-9 plasma levels after intraperitoneal treatment with liposomal Curcumin can constitute valuable markers for evaluating the risk of vascular complications associated with diabetes mellitus. 

According to our results, the liposomal curcumin could constitute a valuable adjuvant therapy in diabetes mellitus due to its effects on glycemia and transaminases changes induced by experimental diabetes mellitus. Moreover, by improving the metalloproteinases level, liposomal curcumin can constitute an important treatment for lowering the risk of vascular complications of diabetes mellitus.

Further larger preclinical and clinical studies are needed to show the relationship between glycemic control, the level of oxidative/anti-oxidant stress markers, and plasma concentration of MMPs and their contribution to vascular complications of DM.

## Figures and Tables

**Table 1:** Variability in oxidative stress, antioxidants, the marker of pancreatic damage, hepatic enzymes and matrix metalloproteinases by groups expressed as mean and standard deviation.

	Group Abbreviation (7 Rats per Group)					
	C	STZ-C	STZ + CC1	STZ + CC2	STZ + lCC1	STZ + lCC2
Oxidative stress						
MDA (nmol/mL)	2.08 (0.31)	20.74 (1.23)	13.50 (1.47)	11.00 (1.23)	6.92 (0.27)	4.52 (0.31)
NO (μmol/L)	26.29 (3.15)	61.29 (2.29)	44.00 (2.94)	42.14 (2.54)	33.71 (2.93)	28.86 (2.61)
TOS (μmol/L)	17.43 (1.72)	72.14 (4.02)	48.86 (1.57)	49.29 (3.04)	42.00 (3.27)	32.29 (2.29)
Antioxidants						
TAC (mEq/L)	1.16 (0.03)	0.73 (0.10)	0.86 (0.10)	0.94 (0.13)	1.10 (0.09)	1.20 (0.03)
Catalase (U/mL)	19.59 (2.40)	11.46 (1.26)	12.51 (1.58)	13.24 (1.45)	15.04 (1.84)	16.03 (1.58)
Marker of pancreatic damage						
Glycemia (mmol/L)	3.42 (0.24)	23.99 (0.47)	22.21 (0.43)	21.49 (0.32)	20.36 (0.37)	18.95 (0.74)
Markers of hepatic damage—hepatic enzymes						
AST (U/L)	33.14 (5.37)	167.71 (8.36)	129.43 (5.26)	116.57 (4.04)	83.14 (3.34)	70.71 (2.63)
ALT (U/L)	34.57 (3.36)	216.43 (5.86)	108.00 (7.14)	95.29 (4.11)	70.86 (4.78)	54.57 (2.51)
Matrix metalloproteinases						
MMP-2 (ng/mL)	86.00 (8.47)	215.71 (10.70)	187.86 (7.76)	174.86 (8.36)	127.29 (5.09)	105.29 (9.05)
MMP-9 (ng/mL)	20.57 (1.62)	34.86 (2.12)	32.00 (1.41)	30.43 (2.37)	27.29 (2.43)	25.00 (1.83)

C = control; STZ-C = streptozotocin control; STZ + CC1 = STZ and 1 mg/100 g bw curcumin as pre-treatment; STZ + CC2 = STZ and 2 mg/100g bw curcumin as pre-treatment; STZ + lCC1 = STZ and pre-treatment with 1 mg/100g bw liposomal-curcumin; STZ + lCC2 = STZ and pre-treatment with 2 mg/100 g bw liposomal-curcumin; MDA = malondialdehyde; NOx = the indirect assessment of NOx synthesis; TOS = total oxidative status; TAC = total antioxidant capacity; AST = aspartate aminotransferase; ALT = alanine aminotransferase.

**Table 2:** Design of experiment on experimental diabetes mellitus with curcumin pre-treatment.

Group (Abbreviation)	Administration Route, Dose [ref]
Control (C)	1 mL i.p. saline solution, 0.9%
Streptozotocin control (STZ-C)	1 mL i.p. STZ * [2,24,100]
STZ and curcumin (1 mg/100 g bw) solution pre-treatment (STZ + CC1)	1 mL i.p. STZ * 1 mL i.p. Curcumin solution, 1 mg/100 g bw
STZ and curcumin (2 mg/100 g bw) solution pre-treatment (STZ + CC2)	1 mL i.p. STZ * 1 mL i.p. Curcumin solution, 2 mg/100 g bw
STZ and liposomal-curcumin (1 mg/100 g bw) solution pre-treatment (STZ + lCC1)	1 mL i.p. STZ * 1 mL i.p. Liposomal-curcumin, 1 mg/100 g bw
STZ and liposomal-curcumin (2 mg/100 g bw) solution pre-treatment (STZ + lCC2)	1 mL i.p. STZ * 1 mL i.p. Liposomal-curcumin, 2 mg/100 g bw

* 60 mg/kg bw.
